# Supplementary material for: Docetaxel Loaded in Copaiba Oil-Nanostructured Lipid Carriers as a Promising DDS for Breast Cancer Treatment
Source: Molecules. 2022 Dec 13;27(24):8838. doi: 10.3390/molecules27248838 (PMC9788038; doi:10.3390/molecules27248838)
Supplement: Supplementary file 1 [file molecules-27-08838-s001.zip › molecules-2068097-supplementary.pdf]

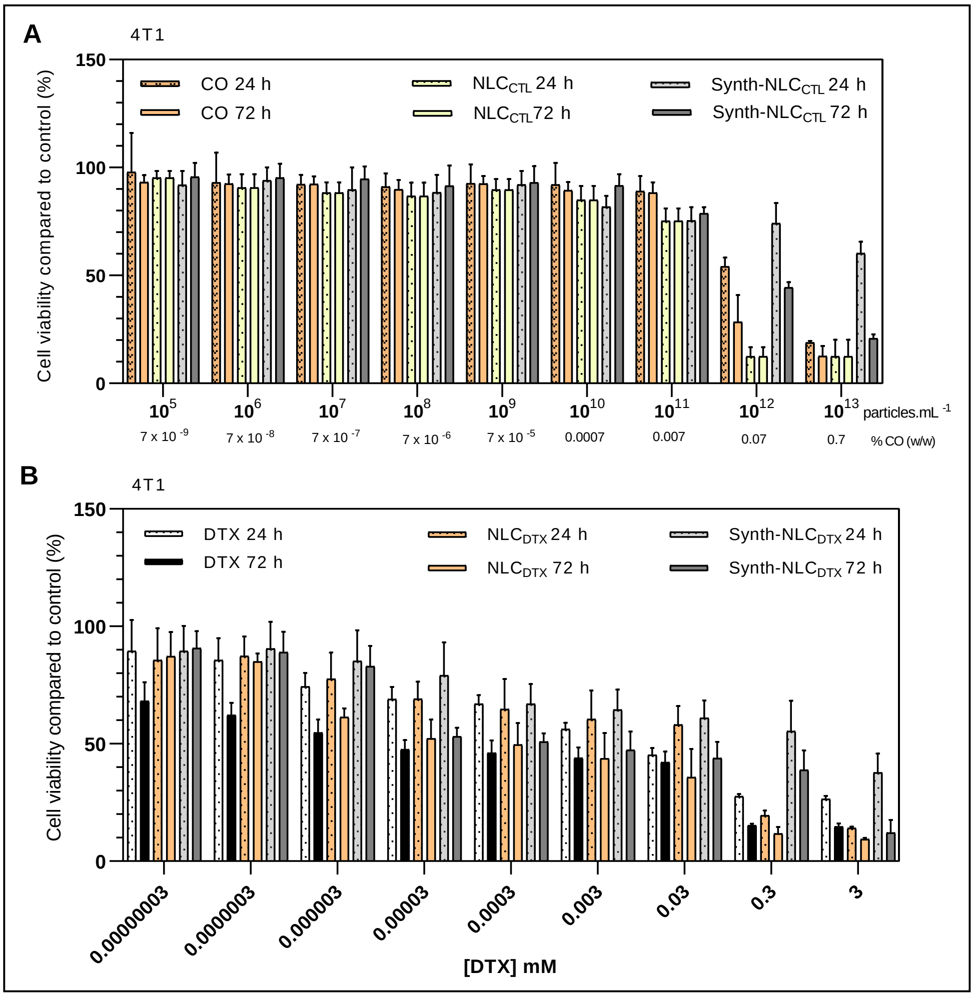

Figure S1. Cell viability (determined by the MTT assay) of 4T1 cells treated for 24 or 72 h with control samples: CO, NLC_{CTL} and Synth-NLC_{CTL} (**A**) and docetaxel-containing samples: DTX, NLC_{DTX} or Synth-NLC_{DTX} (**B**).

Table S1. Design of Experiments. NLC excipients and their interactions with significant effects for the responses (size, PDI and ZP) analyzed in the optimization of NLC formulations prepared with: myristyl myristate, Miglyol 812® + copaiba oil, Pluronic F-68 and docetaxel.

Responses	Range	Positive Effect	Negative Effect
**Size (nm)**	144.1 – 196.8	-	B
**PDI**	0.125 – 0.175	B, C and AC	-
**ZP (mV)**	−23.9 – −27.5	-	-

Abbreviations: PDI = polydispersity index, ZP = Zeta potential. The Capital letters A, B, C refer to the variables tested in DoE experiments: A – LL; B – P68; C – DTX.

Table S2. NTA analysis: Size, dispersion - at 10 % (D10), 50 % (D50), 90 % (D90) of particles size, Span index and particle concentration.mL^{-1} of the optimized formulation and its control (prepared without docetaxel).

Formulation	Size (nm)	Number of particles (x 10^{13}).mL^{-1}	D10 (nm)	D50 (nm)	D90 (nm)	Span*
**NLC$_{DTX}$**	184.2 ± 4.5	4.2 ± 0.3	132.2 ± 3.9	168.8 ± 4.7	253.8 ± 9.5	0.7
**NLC$_{CTL}$**	184.5 ± 3.8	4.1 ± 0.3	136.1 ± 2.6	170.6 ± 3.4	248.8 ± 8.2	0.7

***Calculated according to Li et al.** [78].

Table S3. R^2 coefficients determined for different mathematical models applied to the *in vitro* release kinetic curves of Figure 6, using the KinetDS 3.0 software.

	R^2			
	**Models**			
	**Zero order**	**First order**	**Korsmeyer-Peppas**	**Weibull**
**Commercial DTX**	0.5892	0.1867	0.8755	**0.9522**
**NLC$_{DTX}$**	**0.9486**	0.2458	0.8382	0.8789

Table S4. IC_{50} values determined for DTX (free or encapsulated in NLC$_{DTX}$ or Synth-NLC$_{DTX}$) against 4T1, MCF-7, NIH-3T3 cells, after 24 h of treatment and measured by the MTT assay. Analysis made with the GraphPad Prisma 8 software with the values from the curves in Figure 7.

	**Cancer cell lines**		**Non-cancerous cell line**
	**4T1 (Murine)**	**MCF-7 (Human)**	**NIH-3T3 (Murine)**
**Formulation**	**IC_{50} (μM)**	**IC_{50} (μM)**	**IC_{50} (μM)**
**DTX**	0.84 ± 0.58	32.05 ± 7.91	3.56 ± 3.38
**NLC$_{DTX}$**	7.32 ± 7.07	24.20 ± 28.03	4.21 ± 4.99
**Synth-NLC$_{DTX}$**	3.75 ± 5.44	15.77 ± 51.83	2.99 ± 4.38
